# Tumor Site Immune Markers Associated with Risk for Subsequent Basal Cell Carcinomas

**DOI:** 10.1371/journal.pone.0025160

**Published:** 2011-09-29

**Authors:** Ronald Glaser, Rebecca Andridge, Eric V. Yang, Arwa Y. Shana'ah, Michael Di Gregorio, Min Chen, Sheri L. Johnson, Lawrence A. De Renne, David R. Lambert, Scott D. Jewell, Mark A. Bechtel, Dean W. Hearne, Joel Bain Herron, Janice K. Kiecolt-Glaser

**Affiliations:** 1 Institute for Behavioral Medicine Research, The Ohio State University Medical Center, Columbus, Ohio, United States of America; 2 Department of Molecular Virology, Immunology and Medical Genetics, The Ohio State University Medical Center, Columbus, Ohio, United States of America; 3 Department of Internal Medicine, The Ohio State University Medical Center, Columbus, Ohio, United States of America; 4 Comprehensive Cancer Center, The Ohio State University Medical Center, Columbus, Ohio, United States of America; 5 Division of Biostatistics, The Ohio State University College of Public Health, Columbus, Ohio, United States of America; 6 Division of Pulmonary, Allergy, Critical Care and Sleep Medicine, The Ohio State University Medical Center, Columbus, Ohio, United States of America,; 7 Department of Pathology, The Ohio State University Medical Center, Columbus, Ohio, United States of America; 8 Department of Psychology, University of California, Berkeley, Berkeley, California, United States of America; 9 Division of Dermatology, The Ohio State University Medical Center, Columbus, Ohio, United States of America; 10 Department of Psychiatry, The Ohio State University Medical Center, Columbus, Ohio, United States of America; University Hospital Hamburg-Eppendorf, Germany

## Abstract

**Background:**

Basal cell carcinoma (BCC) tumors are the most common skin cancer and are highly immunogenic.

**Objective:**

The goal of this study was to assess how immune-cell related gene expression in an initial BCC tumor biopsy was related to the appearance of subsequent BCC tumors.

**Materials and Methods:**

Levels of mRNA for CD3ε (a T-cell receptor marker), CD25 (the alpha chain of the interleukin (IL)-2 receptor expressed on activated T-cells and B-cells), CD68 (a marker for monocytes/macrophages), the cell surface glycoprotein intercellular adhesion molecule-1 (ICAM-1), the cytokine interferon-γ (IFN-γ) and the anti-inflammatory cytokine IL-10 were measured in BCC tumor biopsies from 138 patients using real-time PCR.

**Results:**

The median follow-up was 26.6 months, and 61% of subjects were free of new BCCs two years post-initial biopsy. Patients with low CD3ε CD25, CD68, and ICAM-1 mRNA levels had significantly shorter times before new tumors were detected (p = 0.03, p = 0.02, p = 0.003, and p = 0.08, respectively). Furthermore, older age diminished the association of mRNA levels with the appearance of subsequent tumors.

**Conclusions:**

Our results show that levels of CD3ε, CD25, CD68, and ICAM-1 mRNA in BCC biopsies may predict risk for new BCC tumors.

## Introduction

In 2006, more than 2 million individuals in the United States were treated for non-melanoma skin cancers, mostly basal cell carcinomas (BCC) [1], [2]. Studies have linked a number of risk factors to onset of BCCs, including male gender, fair skin that burns easily and tans poorly, red or blond hair, blue eyes, Celtic ancestry, proximity to the equator, older age, occupations that involve sun or arsenic exposure, cumulative benign sun-related skin damage, and sunburns before the age of 18 [3]. Indeed, sun exposure during childhood may be particularly important in determining risk [4]. The factors influencing the risk for additional primary BCCs after the first are not completely clear.

There is good evidence that BCC is an immunogenic tumor. For example, patients who are immunosuppressed following organ transplantation have a substantially elevated risk for new BCCs, as well as squamous cell carcinoma [5], [6], [7], [8]. Comparisons of BCC patients with sociodemographically-matched controls show poorer immune responses among the former, including poorer proliferative responses to the mitogens concanavalin A (Con A) and phytohemagglutinin (PHA), as well as decreased responsiveness of T-cells to antigens, such as *Candida*. Furthermore, BCC tumors that regress are characterized by greater lymphocyte trafficking to the tumor and the surrounding stroma compared to tumors that do not regress [9], [10], [11], [12]. In one study the authors compared the cells trafficking to regressing and non-regressing BCCs and found that regressing BCCs had a large number of CD4+ T lymphocytes, but not CD8+ lymphocytes. In addition, the numbers of interleukin (IL)-2 receptor positive T lymphocytes and transferrin receptor-positive T lymphocytes were greater in regressing tumors compared to those that were not regressing, indicating that the T cells were activated [9], [10]. Fifty percent of tumors provide evidence of at least partial regression [10], [13]. The risk for development of subsequent primary BCCs after an initial lesion is substantial, with 44% developing additional lesions within 3 years [14].

Further evidence for the critical role of the immune response in BCC tumor development is the efficacy of imiquimod in the treatment of this disease. This topical cream stimulates a local immune response to BCC tumor cells. Imiquimod-induced regression of BCC tumors was associated with increased infiltration of inflammatory cells (within 3 to 5 days after initiation of imiquimod treatment) concomitant with enhanced expression of the intercellular adhesion molecule (ICAM)-1 [15]. Furthermore, comparisons of the inflammatory infiltrates of BCC tumors pre- and post-imiquimod treatment have shown that the predominant infiltrating inflammatory cells were CD3+ and CD8+ T-cells, although CD68+ cells (macrophages) also increased [16]. These data are in accord with studies of regressing BCCs that showed elevated levels of interferon gamma (IFN-γ), IL-2 (a T-cell marker), and CD3ε (a T-cell cell surface marker) as measured by RT-PCR, indicating an enhanced antitumor Th1 immune response[10]. Thus, the expression of several immune-related markers has been implicated in BCC progression [11], [12]; and we based our selection of markers on these studies. We measured CD3ε, IFN-γ, CD25, CD68, ICAM-1 and IL-10 mRNA expressed in BCC tumors to assess the relationship between the expression of these genes (as measured by real time PCR) and the occurrence of subsequent BCC tumors.

## Materials and Methods

### Participants

The BCC patient pathology results were obtained from dermatology outpatient clinics affiliated with the Ohio State University Medical Center. The final 138 BCC patients in our study were screened for immunosuppressive disorders and immunosuppressive drugs. They were not on medications that would have promoted skin cancer. For those cases with tissue samples of usable size, patient electronic medical records were used to exclude individuals with immunosuppressive therapies, immunological treatments for other medical conditions, another cancer diagnosed within the last five years (except for a prior BCC), or any history of SCC or melanoma. Eligible patients received a letter from their treating dermatologist describing the study; those who responded were contacted by phone to verify medical history and study eligibility. A total of 174 participants met eligibility criteria and were enrolled in the study. Of these, 7 did not have sufficient mRNA, 27 did not provide follow-up data, and 2 were missing both mRNA and follow-up data. This left an analysis sample of 138 BCC patients. Subjects excluded from the analysis did not differ significantly from included subjects on any demographic or clinical characteristics. The Ohio State University Biomedical Research Review Committee approved the project; all subjects gave written informed consent prior to participation.

### Follow-up and endpoints

Patients were mailed questionnaires every six months for a maximum of three years after the biopsy date of the BCC tumor used in study analyses. The median follow-up was 26.6 months [17]. At each assessment point, patients reported the month and year of any new BCC removals that had occurred within the previous six months. They also indicated whether or not they had any other type of skin cancer, and they listed any changes in their medical condition or current medications. Follow-up BCC pathology data were also verified through the OSU Medical Center electronic medical records both to validate patient self-report data and identify any additional BCC tumors not reported by patients. BCC tumor-free time period was defined as the time from the biopsy date of the BCC tumor to the appearance of the first subsequent new primary BCC tumor. The censoring date for participants free of new tumors was the last contact date.

### RNA extraction and cDNA synthesis

Four sections (10 µm thick each) were obtained from each of the biopsies. The sections included tumor and surrounding area that included some normal skin, dermis and lymphocytic infiltrate. The paraffin-embedded diagnostic biopsies were combined in an Eppendorf tube and immediately deparaffinized with xylene followed by hydration through graded ethanol washes. After the pathology lab prepared the four sections an additional section was obtained in order to confirm that the four tissue sections were still positive for tumor tissue. The tissue was then centrifuged and digested with 100 µl digestion buffer (0.01 M Tris pH7.8, 0.005 M EDTA, and 0.5% SDS) plus 100 µl of 20 mg/ml proteinase K for 24 hours at 55°C with agitation. Total RNA was extracted by adding 800 µl Trizol Reagent (Life Technologies) with 200 µg/ml glycogen as a carrier. Total RNA was precipitated with 650 µl of 100% isoproponol at –40°C overnight then centrifuged at maximum speed at room temperature. The pellets were washed twice with 75% ice-cold ethanol. The RNA pellet was resuspended in nuclease-free H_2_O, and quantified using a Nanodrop spectrophotometer. Total RNA (1 µg) was treated with DNase I (Life Technologies), followed by cDNA synthesis using Superscript III RNase H- reverse transcriptase (Life Technologies). cDNA was stored at −80°C until used for real-time PCR.

### Gene Expression Studies, Real-time PCR and Data Analysis

TaqMan Gene Expression Assays (Applied Biosystems) were used for both internal positive controls and the genes of interest. Using the Taqman Human Endogenous Control Plates (Applied Biosystems) and the GeNorm software (http://medgen.ugent.be/~jvdesomp/genorm/) we selected the following as the appropriate internal positive controls for this study: GAPDH (glyceraldehydes-3-phosphate-dehydrogenase; Assay ID: 4326317E) and RPLP0 (large ribosomal protein; Assay ID 4326314E). The levels of expression of CD3ε (Assay ID: Hs99999153_m1), CD25 (Assay ID: 4328847F), CD68 (Assay ID: Hs00154355_m1), ICAM-1 (Assay ID: Hs99999152_m1), IFN-γ (Assay ID: Hs99999041_m1) and IL-10 (Assay ID: Hs99999035_m1) mRNAs were normalized to the geometric average of the C_T_s for GAPDH and RPLP0. TaqMan Gene Expression Assay Reagents do not detect genomic DNA sequences; therefore, these are specific to mRNA. mRNA levels between samples were compared using relative real-time RT-PCR with TaqMan fluorogenic probes, TaqMan PCR Reagent Kit and 7300 Real-Time PCR System (Applied Biosystems). We also included one sample in all of the PCR runs to serve as our positive plate control. All genes of interest were first normalized to the internal positive control, then normalized to the positive plate control, and the relative expression of mRNA species was calculated using the comparative C_T_ method as described by the manufacturer (see User bulletin #2 Applied Biosystems, P/N 4303859, 1997) [18].

### Statistical Methods

Univariate linear regression analyses and ANOVA models were used to determine whether mRNA variables (log-transformed) were significantly associated with demographic and clinical characteristics. Kaplan-Meier plots and Cox proportional hazards were used for survival analyses. The proportional hazards assumption was checked for all predictive factors for the Cox models, and, when the assumption was found to have failed, interactions with time were incorporated to address the nonproportional hazards. Initially, univariate analyses examining the effect of mRNA variables on tumor-free time period were performed, using a cutpoint of the median observed value for each marker. Multivariate analyses were performed, controlling for age and gender, as well as significant interactions. We conducted parallel analyses using the log-transformed mRNA markers values; resulting conclusions were similar and are not shown. Alpha was set to 0.05, and two-sided tests were conducted. All analyses were performed using SAS version 9.1.

## Results

### Patient characteristics

Demographic and clinical characteristics of the 138 subjects who had sufficient mRNA and follow-up data are summarized in Table 1. The average age was 57.9 years (SD = 13.2, range = 23–92. Forty-eight subjects had nodular BCC, 35 had superficial BCC, and the remaining 55 had mixed tumors. Half of the subjects had tumors removed from the head and neck area (n = 69). Sun exposure and sunburn history varied widely and is summarized in Table 1 and Table S1.

**Table 1:** Demographic and clinical characteristics of the analysis sample (n = 138).

Characteristic				
Demographics				
	Female sex, No. (%)		71	(51%)
	White race, No. (%)		137	(99%)
	Age (years), Mean (SD)		57.9	(13.2)
	Age group (years), No. (%)			
		<30	3	(2%)
		30–39	9	(7%)
		40–49	21	(15%)
		50–59	46	(33%)
		60–69	38	(28%)
		70–79	14	(10%)
		80+	7	(5%)
	Education, No. (%)			
		High school or less	26	(19%)
		Some college	79	(57%)
		College degree	33	(24%)
	Smoking status, No. (%)			
		Current	10	(7%)
		Former	50	(36%)
		Never	77	(56%)
		Unknown	1	(1%)

Clinical Characteristics				
	BCC tumor type, No. (%)			
		Nodular	48	(35%)
		Superficial	35	(25%)
		Mixed type	55	(40%)
	Had a prior BCC, No. (%)		111	(80%)
	Number of BCC removed, No. (%)			
		Single	101	(73%)
		Multiple	19	(14%)
		Unknown	18	(13%)
	BCC site, No. (%)			
		Head and neck	69	(50%)
		Trunk	30	(22%)
		Upper limbs	22	(16%)
		Lower limbs	6	(4%)
		Multi-site	7	(5%)
		Unknown	4	(3%)

Sun exposure / Skin Type				
	Skin type - Burning			
		Always burn	15	(11%)
		Usually burn	27	(20%)
		Burn moderately	47	(34%)
		Burn minimally	30	(22%)
		Rarely/never burn	19	(14%)
	Number of sunburns before age 19			
		0	11	(8%)
		1–9	55	(40%)
		10–19	41	(30%)
		20+	30	(22%)
		Unknown	1	(1%)

Additional sun exposure characteristics are available in Table S1.

Questions from the Older Adults Resources Survey (OARS) Multidimensional Functional Assessment Questionnaire provided data on the frequency of chronic conditions in the study population [19] . Among the final 138 participants, the following comorbid conditions were reported either at baseline or during follow-up periods: arthritis, n = 15 (11%), asthma n = 6 (4%), diabetes, n = 3 (2%), emphysema, n = 2 (1%), heart disease, n = 21 (15%), hypertension, n = 61 (44%), kidney disease, n = 2 (1%), liver disease, n = 1 (1%), stroke, n = 7 (5%) and thyroid disease, n = 9 (7%).

### Association between mRNA markers and patient characteristics

CD3ε, CD25, and ICAM-1 mRNA levels in tumor biopsies showed a significant correlation with histological tumor type (p = 0.003, p = 0.004, p = 0.03, respectively), with nodular tumor type having lower levels of gene expression than the superficial tumors or mixed tumors. Tumors removed from the head and neck had lower levels of CD3ε, CD25, ICAM-1, and IFN-γ mRNA compared to other sites (p = 0.002, p = 0.003, p = 0.01, p = 0.03, respectively). Patients with a history of a prior BCC had significantly lower levels of CD3ε, CD25, CD68, and ICAM-1 mRNA (p = 0.01, p = 0.01, p = 0.005, p = 0.03, respectively). There were no associations between mRNA levels and the sun exposure variables. None of the mRNA markers were significantly related to demographic factors (age, gender, education level, smoking status). Though the number of patients with comorbid conditions was small, we did observe that patients with asthma (n = 6) had lower levels of IL-10 mRNA (p = 0.05); there were no associations between mRNA levels and other autoimmune related diseases (diabetes, arthritis).

### Associations among mRNA markers

Strong correlations existed among immune cell-associated mRNA markers (Table 2). CD25, CD68, and ICAM-1 were all highly positively correlated with CD3ε (r>0.8 for all three markers, p<0.0001). The strength of the association of IL-10 and IFN-γ with CD3ε was lower, r = 0.67 and r = 0.24 respectively, though still statistically significant (p<0.001 for both). Thus, similar patterns of association with tumor-free periods emerged for many immune cell markers, as described below.

**Table 2:** Correlations (n) between pairs of mRNA markers.

	CD25	CD68	ICAM-1	IFN-γ	IL-10
CD3ε	**0.92**	**0.84**	**0.80**	**0.24**	**0.67**
	(136)	(136)	(135)	(136)	(79)
CD25		**0.88**	**0.84**	**0.17**	**0.75**
		(136)	(135)	(136)	(79)
CD68			**0.81**	0.09	**0.77**
			(136)	(137)	(79)
ICAM-1				0.13	**0.77**
				(136)	(79)
IFN-γ					**0.24**
					(79)

P-values<0.05 in bold. All mRNA values are log-transformed.

### Overall tumor-free time period and association with patient demographics

Overall 61% of subjects were free of new BCCs at two years post-initial biopsy (95% CI: 53%-70%). There was a borderline effect of gender on risk for subsequent tumors; two years after the initial biopsy 54% of males and 70% of females were free of new BCCs (log-rank p = 0.12). There was a significant effect of age that increased over time (p = 0.01), with older age increasing the risk of a subsequent tumor. Therefore all adjusted models included gender, age and an interaction between age and time. Patients who had multiple BCCs removed had a marginally higher risk of a subsequent tumor; at two years post-biopsy, 44% of subjects with multiple BCCs removed were free of subsequent tumors compared to 65% of subjects with a single BCC removed (log-rank p = 0.08). Neither type of BCC tumor nor history of prior BCC tumors was significantly associated with time to subsequent tumor (log-rank p = 0.61, p = 0.59, respectively). No other demographic or sun exposure variables listed in Table 1 were significantly associated with time to subsequent BCCs.

**Figure 1:**
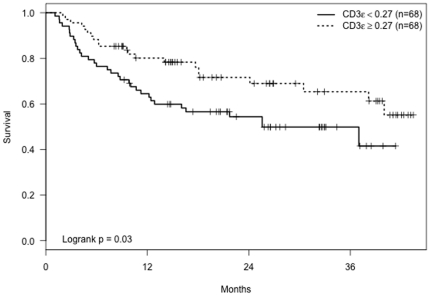
Kaplan-Meier curves showing tumor-free time period for patients with CD3ε mRNA levels ≥and<0.27 (the median CD3ε value).

**Figure 2:**
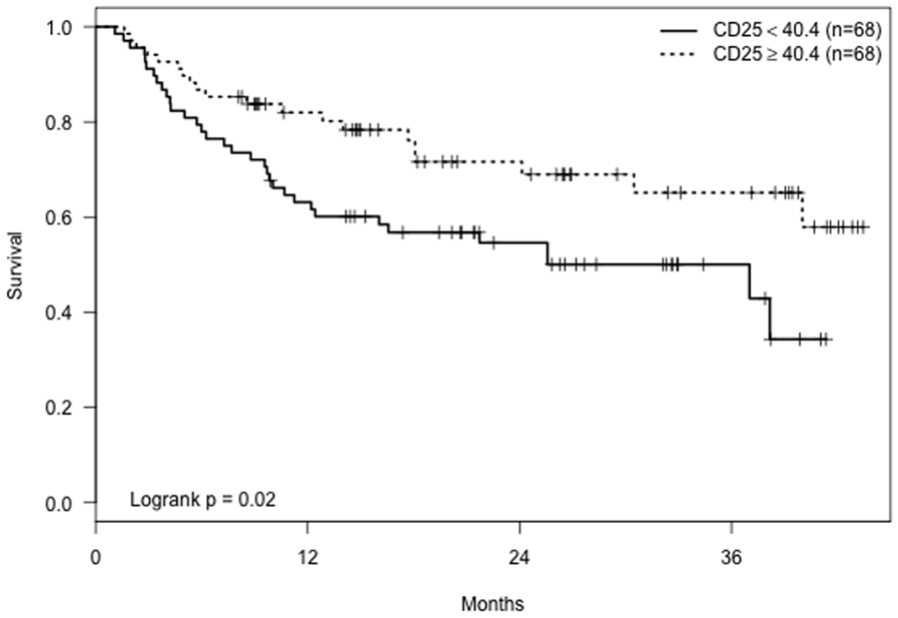
Kaplan-Meier curves showing tumor-free time period for patients with CD25 mRNA levels ≥and<40.4 (the median CD25 value).

Univariate survival analyses using median cutpoints for immune cell markers revealed that patients with low CD3ε mRNA levels in their tumor biopsies had significantly shorter tumor-free time periods (p = 0.03, Figure 1). Patients with low CD25 mRNA levels also had significantly shorter tumor-free periods (p = 0.02, Figure 2). There was no significant association between IFN-γ levels and time until subsequent tumors (p = 0.21). Adjusted analyses revealed that older age dampened the effect of low levels of CD3ε and CD25 cells on time to subsequent tumors (Table 3). The estimated relative risk of subsequent new tumors for low versus high CD3ε cells was 2.6 for subjects aged 50 at initial BCC removal (p = 0.01), but this hazard ratio declined to 1.8 (p = 0.04) for subjects aged 60 and 1.2 (p = 0.53) for subjects aged 70. Similarly, the hazard ratio for low compared to high CD25 was higher for younger ages but declined to nonsignificance by age 70 (Table 3).

**Table 3:** Tumor-free time period: Hazard ratios (HR) for low versus high levels of immune markers.

Models†			Unadjusted model				Adjusted		
Model	N	Comparison	HR	95% CI	p-value	Age*	HR	95% CI	p-value
CD3ε	136	low vs. high	1.9	1.1–3.2	**0.03**	50	2.6	1.2–5.7	**0.01**
						60	1.8	1.03–3.2	**0.04**
						70	1.2	0.63–2.4	0.53
						*Age x CD3*ε *interaction p = 0.10*			
IFN-γ	138	low vs. high	0.71	0.42–1.2	0.21	any	0.73	0.42–1.2	0.24
CD25	136	low vs. high	1.9	1.1–3.3	**0.02**	50	3.2	1.4–7.2	**0.004**
						60	1.9	1.04–3.3	**0.04**
						70	1.1	0.55–2.1	0.84
						*Age x CD25 interaction p = 0.02*			
CD68	137	low vs. high	2.3	1.3–4.2	**0.004**	50	3.9	1.7–8.7	**0.001**
						60	2.3	1.29–4.2	**0.005**
						70	1.4	0.70–2.8	0.34
						*Age x CD68 interaction p = 0.04*			
ICAM-1	136	low vs. high	1.6	0.94–2.8	0.08	50	2.3	1.1–4.9	**0.04**
						60	1.6	0.91–2.8	0.11
						70	1.1	0.57–2.2	0.75
						*Age x ICAM-1 interaction p = 0.12*			
IL-10	79	low vs. high	1.6	0.82–3.2	0.16	any	1.8	0.87–3.7	0.12
Score**	135	1–3 vs. 0	2.8	1.3–5.9	**0.008**	50	4.5	1.5–14	**0.009**
						60	2.6	1.2–6	**0.02**
						70	1.5	0.6–3.5	0.38
		4 vs. 0	2.5	1.2–5.4	**0.02**	50	4.2	1.4–12.9	**0.01**
						60	2.4	1.09–5.5	**0.03**
						70	1.4	0.60–3.4	0.42

Separate Cox models for each biomarker.

† Adjusted for gender, age at initial BCC, interaction of age with time.

*Some models have an interaction of biomarker level (binary) with age at initial BCC removal (continuous variable), as shown above. When the interaction was present, the effect of mRNA marker level is interpreted for a specific age.

**Combined score defined as the number of mRNA markers out of CD3ε, CD25, CD68, and ICAM-1 that are below the median: 0 (none below) / 1–3 (some below) / 4 (all below). P-value for age by score interactions are p = 0.07 and p = 0.08 for some vs. none and all vs. none, respectively.

Effects are presented separately for age 50, 60, and 70 where age moderated effects.

**Figure 3:**
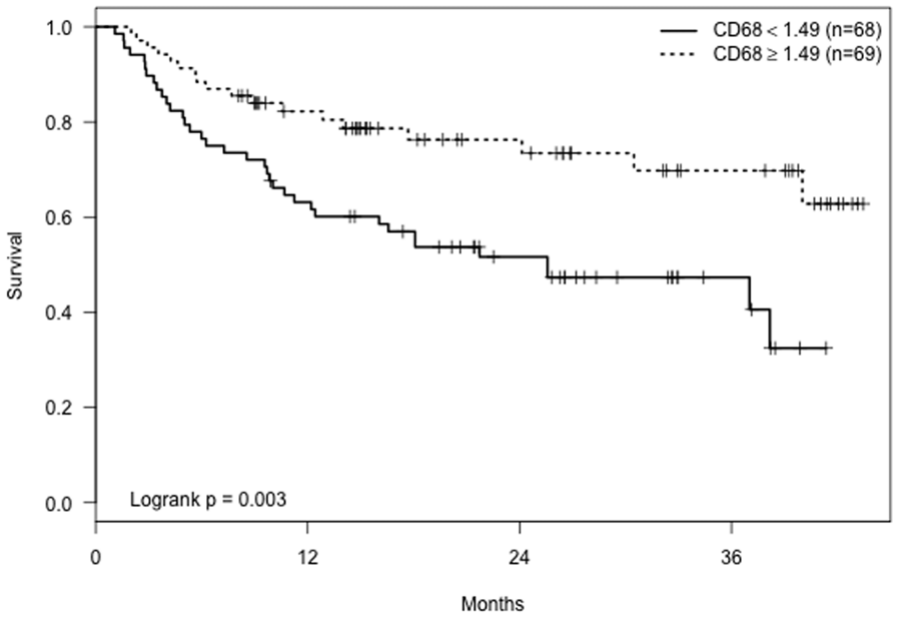
Kaplan-Meier curves showing tumor-free time period for patients with CD68 mRNA levels≥and <1.49 (the median CD68 value).

Without adjusting for age or gender, analyses showed that patients with low CD68 mRNA expression in biopsies had significantly shorter tumor-free periods (p = 0.003, Figure 3). In adjusted models (Table 3) the effect of CD68 was modified by age, similar to CD3ε and CD25. The risk of recurrence for low versus high CD68 was high for younger subjects (e.g., HR = 3.9, p = 0.004 for a subject aged 50) but declined with increasing subject age (e.g., HR = −1.1, p = 0.84 for a subject aged 70).

Low ICAM-1 mRNA levels in tumor biopsies had a borderline association with shorter tumor-free periods (p = 0.08). In adjusted models (Table 3), only at younger ages was the effect of low ICAM-1 mRNA levels significant as older age decreased the effect of the biomarker. There were no significant adjusted or unadjusted associations between IL-10 mRNA levels and the appearance of subsequent tumors. However, only 79 of 138 patients had IL-10 mRNA measurements available, reducing the power to detect an effect. Nonetheless, effects were in the same direction as the other mRNA markers, with low IL-10 levels associated with an increased risk of subsequent tumors (adjusted HR = 1.8, p = 0.12).

**Figure 4:**
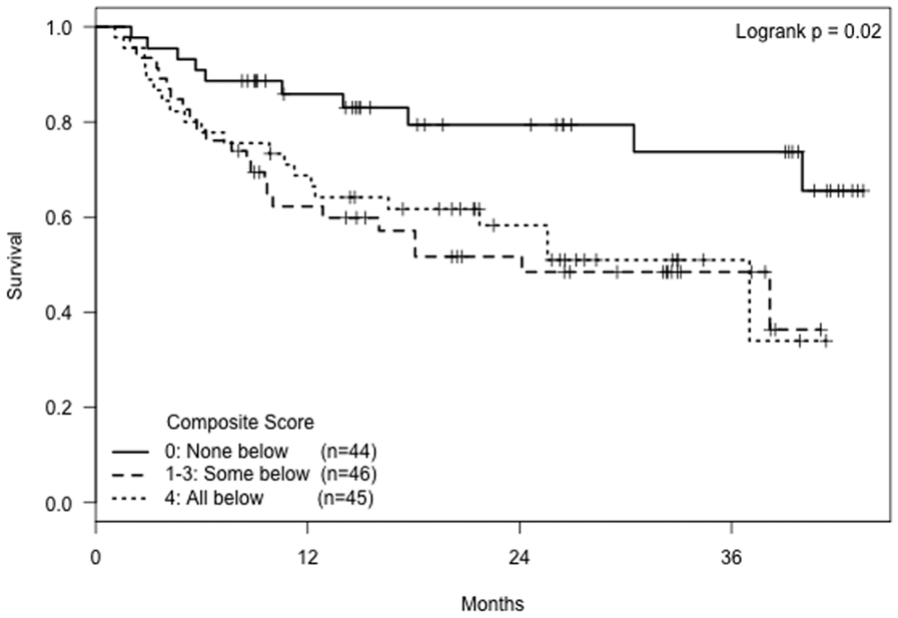
Kaplan-Meier curves showing tumor-free time period by composite mRNA marker score, defined as the number of mRNA markers (CD3ε, CD25, CD68, and ICAM-1) that are below the median.

A composite score using the four significant mRNA markers (CD3ε, CD25, CD68, and ICAM-1 mRNA) was generated (n = 135 who had all markers available). The score was equal to the number of markers that were below the median observed value (ranging from 0 to 4). Thirty-three percent of subjects (n = 44) had a score of zero, indicating high expression of all four markers. Another 34% (n = 46) had scores ranging from one to three, indicating elevated expression of some but not all markers. The final 33% of subjects (n = 45) had below-median levels of all four markers. Univariate analyses using this composite score (Figure 4) revealed that patients with a 0 score had a statistically significantly longer tumor-free time period compared to subjects with a score of 4 (HR = 0.40, p = 0.02) or a score of 1-3 (HR = 0.36, p = 0.008). There was no significant difference between the recurrence-free time period of subjects with a score of 4 compared to those with scores 1–3 (p = 0.75). Multivariate analyses adjusting for age and gender showed results similar to those of the individual markers with an interaction of age with score (Table 3).

To investigate potential confounding, clinical characteristics that were significantly associated with either mRNA levels or subsequent BCC occurrence were added to the survival models describing the effect of mRNA levels. Controlling for these characteristics did not substantially change point estimates or change overall conclusions. In addition, all such variables were not significant in the adjusted models and thus were left out of the final presentation of results.

## Discussion

Patients who have had a previous BCC are at increased risk for new primary BCCs [14]. Our study investigated the expression of several immune-related biomarkers (i.e., CD3ε, CD25, CD68, ICAM-1, IFN-γ, and IL-10) in the peri-tumoral milieu of BCC tumors and their relationship to the appearance of subsequent new BCCs. We found that lower levels of CD3ε, CD25, CD68, and ICAM-1 mRNA in BCC tumor biopsies at baseline predicted subsequent BCCs.

Prior studies have characterized the BCC peri-tumoral milieu. For example, Hunt and co-workers studied primary BCC tumors with and without histologic evidence of active or past regression. Regression was defined histologically as the disruption of the normal outline of tumor islands accompanied by lymphocytic infiltration penetrating and surrounding the tumor [13]. They suggested that some BCC tumors induced immune responses that could lead to evidence of tumor disruption. In addition, they found a significant increase in CD3 (a measure of total T cells) in actively regressing tumors compared with those showing no regression. Finally, expression of CD25 (IL-2 receptor) was greater in the actively regressing BCC tumors compared with tumors that showed no evidence of current or past regression. Consistent with their data, we found that lower CD3ε and CD25 mRNA levels in BCC tumors were associated with increased risk for developing subsequent BCCs.

In addition, Wong and co-workers described the cytokine profiles of BCC tumors showing histologic regression [10]. They noted a trend towards higher CD3 and IL-2 mRNA levels in actively regressing tumors. Furthermore, IFN-γ mRNA levels were strongly correlated with levels of CD3 mRNA, which was interpreted as evidence for activated lymphocytes producing a Th1 type immune response in actively regressing BCC tumors. However, in contrast to their observation we did not observe an association between IFN-γ mRNA levels and risk for subsequent tumors.

Increased expression of ICAM-1, the ligand for lymphocyte function-associated antigen-1 (LFA-1) on both T- and B-lymphocytes, is one effect of IFN-γ that is linked to anti-tumor properties. For example, imiquimod-induced regression of BCC tumors was associated with increased infiltration of inflammatory cells (within 3 to 5 days after initiation of imiquimod treatment) concomitant with enhanced expression of ICAM-1 [15]. Another study showed that daily treatment of 12 BCCs in 11 patients with 5% imiquimod cream at night for at least 8 h five times per week resulted in the greater influx of inflammatory cells (primarily CD45+ lymphocytes and CD68+ macrophages) in the tumor biopsies compared to biopsies collected before treatment [16]. We found that lower levels of ICAM-1 mRNA were associated with an increased risk of subsequent BCCs. Lower levels of ICAM-1 mRNA could also reflect lower infiltration of inflammatory cells which could enhance risk for subsequent tumors. Our study demonstrated an association between CD3ε, CD25, CD68, and ICAM-1 mRNA levels in BCC tumor biopsies and the risk for subsequent tumors. These results support an important role for inflammatory cells in the peri-tumoral milieu and for immune cells that infiltrate BCC tumors and thus mediate BCC tumor progression [12], [13], [20].

It is interesting to note that we did not observe a significant association between IL-10 mRNA levels and the appearance of subsequent tumors but the trend is as seen with the other markers. Besides being produced by T-cells, B-cells, and monocytes, IL-10 has been shown to be produced by BCC tumor cells as well [11], [21]. This lack of association may be explained by the fact that the methodology used here can detect IL-10 mRNA from both infiltrating immune cells and the BCC tumor cells. The association might have been stronger if we had been able to differentiate the IL-10 mRNA expressed by immune cells from that made by the tumor cells.

Animal models of BCC have demonstrated that BCC growth is impacted by cell mediated, humoral mediated and NK mediated inflammation [22]. Mechanistically, it would have been useful to know if our immune markers were associated with the host immune response as well as with risk of additional BCCs [12], a limitation of this study and an important direction for future work. In addition, future studies should also compare BCC edge mRNA results with adjacent normal skin to support the contention that the mRNA data are most likely due to the immune response and not the circulating inflammatory cells.

The occurrence of BCC can also indicate other cancer risks: individuals with a history of nonmalignant skin cancers are at increased risk for death from a variety of noncutaneous cancers [23], [24], [25], [26]. For example, a large prospective study with over one million participants found mortality over the next 12 years from all noncutaneous cancers was 20 to 30 % higher among individuals who reported no cancer at baseline (other than a history of nonmalignant skin cancer) than among those who did not share the same history; the relative risk for mortality from other cancer was 1.30 in men and 1.26 in women [23]. Indeed, skin cancer patients are at higher risk for second primary non-cutaneous malignancies, often those related to smoking or lymphoproliferative malignancies [24], [25], [26]. Thus, studying factors associated with the BCC risk may also shed some light on broader cancer risks.

Chronic ultraviolet radiation (UVR) exposure leads to the accumulation of elastotic material [27], a potent immunosuppressant [28] that stimulates local production of immunosuppressive molecules [29], which can induce antigen-specific tolerance [30], abrogating the effector phase of cell-mediated immunity [31]. The acquisition of solar elastotic material also disrupts lymphatic drainage, which may also play a role in decreased immunity [32]. We found that tumors removed from the head and neck expressed lower levels of CD3ε, CD25, ICAM-1, and IFN-γ mRNA compared to tumors from other sites. The expression of these markers in tumors from less exposed parts of the body may be related to chronic UVR exposure or deposition of elastotic material.

In this study, we focused on a selection of immune cell markers that have been related to the regression of BCC tumors in previous studies. Recent studies have described the molecular mechanisms involved in the initiation and progression of BCC. These observations have implicated the hedgehog signaling pathway in the molecular pathogenesis of BCC [33], [34]. The pivotal roles of signaling proteins including patched homolog 1 (PTCH-1) and smoothened (SMO) are of great interest to us but this topic is beyond the scope of this study [35]. Future studies could include other immune cell markers such as IL-2 receptor positive cells, dendritic cells and measures of the response of peripheral blood leukocytes to Con A and PHA. Nevertheless, we believe that the data obtained in this study may provide a useful approach for identifying BCC patients at risk for subsequent tumors.

## Supporting Information

Table S1 **Additional sun exposure characteristics of the analysis sample (n = 138).**
(DOC)

## References

[1] American Cancer Society (2010). Cancer Facts & Figures 2010..

[2] Rogers HW, Weinstock MA, Harris AR, Hinckley MR, Feldman SR (2010). Incidence estimate of nonmelanoma skin cancer in the United States, 2006.. Arch Dermatol.

[3] Kiiski V, de Vries E, Flohil SC, Bijl MJ, Hofman A (2010). Risk factors for single and multiple basal cell carcinomas.. Arch Dermatol.

[4] Kricker A, Armstrong BK, English DR (1994). Sun exposure and non-melanocytic skin cancer.. Cancer Causes and Control.

[5] Fortina AB, Piaserico S, Caforio ALP, Abeni D, Alaibac M (2004). Immunosuppressive level and other risk factors for basal cell carcinoma and squamous cell carcinoma in heart transplant recipients.. Arch Dermatol.

[6] Grinyo J (2010). Malignancy after renal transplantation: the role of immunosuppression.. Nat Rev Nephrol.

[7] Naldi L, Boschiero L, Nacchia F, Fior F, Forni A (2010). Incidence and Clinical Predictors of a Subsequent Nonmelanoma Skin Cancer in Solid Organ Transplant Recipients With a First Nonmelanoma Skin Cancer A Multicenter Cohort Study.. Arch Dermatol.

[8] Edelbroek J, de Fijter J, Haasnoot G, Claas F, Willemze R (2010). Subsequent Squamous- and Basal-Cell Carcinomas in Kidney-Transplant Recipients After the First Skin Cancer: Cumulative Incidence and Risk Factors.. Transplantation.

[9] Halliday GM, Patel A, Hunt MJ, Tefany FJ, Barnetson RS (1995). Spontaneous regression of human melanoma/nonmelanoma skin cancer: association with infiltrating CD4+ T cells.. World J Surg.

[10] Wong DA, Bishop GA, Lowes MA, Cooke B, Barnetson RS (2000). Cytokine profiles in spontaneously regressing basal cell carcinomas.. Brit J Dermatol.

[11] Domingo DS, Baron ED (2008). Melanoma and nonmelanoma skin cancers and the immune system.. Advances in experimental medicine and biology.

[12] Kaur P, Mulvaney M, Carlson JA (2006). Basal cell carcinoma progression correlates with host immune response and stromal alterations: a histologic analysis.. Am J Dermatopathol.

[13] Hunt MJ, Halliday GM, Weedon D, Cooke BE, Barnetson RS (1994). Regression in basal cell carcinoma: an immunohistochemical analysis.. Brit J Dermatol.

[14] Marcil I, Stern RS (2000). Risk of developing a subsequent nonmelanoma skin cancer in patients with a history of nonmelanoma skin cancer.. Arch Dermatol.

[15] Urosevic M, Maier T, Benninghoff B, Slade H, Burg G (2003). Mechanisms underlying imiquimod-induced regression of basal cell carcinoma in vivo.. Arch Dermatol.

[16] De Giorgi V, Salvini C, Chiarugi A, Paglierani M, Maio V (2009). In vivo characterization of the inflammatory infiltrate and apoptotic status in imiquimod-treated basal cell carcinoma.. Int J Dermatol.

[17] Schemper M, Smith TL (1996). A note on quantifying follow-up in studies of failure time.. Control Clin Trials.

[18] Livak KJ, Schmittgen TD (2001). Analysis of relative gene expression data using real-time quantitative PCR and the 2(-Delta Delta C(T)) Method.. Methods.

[19] Fillenbaum GG, Smyer MA (1981). The development, validity, and reliability of the OARS multidimensional functional assessment questionnaire.. J Gerontol.

[20] Schlecker C, Pierer G, Haug M (2009). Spontaneous regression of two giant basal cell carcinomas in a single patient after incomplete excision.. Tumori.

[21] Kim J, Modlin R, Moy R, Dubinett S, McHugh T (1995). IL-10 production in cutaneous basal and squamous cell carcinomas. A mechanism for evading the local T cell immune response.. J Immunol.

[22] Carlson JA, Combates NJ, Stenn KS, Prouty SM (2002). Anaplastic neoplasms arising from basal cellcarcinoma xenotransplants into SCID-beige mice.. J Cutan Pathol.

[23] Kahn HS, Tatham LM, Patel AV, Thun MJ, Heath CW (1998). Increased cancer mortality following a history of nonmelanoma skin cancer.. JAMA.

[24] Cantwell MM, Murray LJ, Catney D, Donnelly D, Autier P (2009). Second primary cancers in patients with skin cancer: a population-based study in Northern Ireland.. Br J Cancer.

[25] Nugent Z, Demers AA, Wiseman MC, Mihalcioiu C, Kliewer EV (2005). Risk of second primary cancer and death following a diagnosis of nonmelanoma skin cancer.. Cancer Epidemiol Biomarkers Prev.

[26] Rosenberg CA, Greenland P, Khandekar J, Loar A, Ascensao J (2004). Association of nonmelanoma skin cancer with second malignancy.. Cancer.

[27] Uitto J (2008). The role of elastin and collagen in cutaneous aging: intrinsic aging versus photoexposure.. J Drugs Dermatol.

[28] Kripke ML (1994). Ultraviolet radiation and immunology: something new under the sun–presidential address.. Cancer Res.

[29] Slominski A (2007). A nervous breakdown in the skin: stress and the epidermal barrier.. J Clin Invest.

[30] Schwarz T (1999). Ultraviolet radiation-induced tolerance.. Allergy.

[31] Schwarz A, Maeda A, Wild MK, Kernebeck K, Gross N (2004). Ultraviolet radiation-induced regulatory T cells not only inhibit the induction but can suppress the effector phase of contact hypersensitivity.. J Immunol.

[32] Paul J (2011). Lymphangiectases are common underlying warts and in normal peritumoral skin: histologic evidence of decreased immune surveillance.. Am J Dermatopath.

[33] Barnes EA, Heidtman KJ, Donoghue DJ (2005). Constitutive activation of the shh-ptc1 pathway by a patched1 mutation identified in BCC.. Oncogene.

[34] Hutchin ME, Kariapper MST, Grachtchouk M, Wang A, Wei L (2005). Sustained Hedgehog signaling is required for basal cell carcinoma proliferation and survival: conditional skin tumorigenesis recapitulates the hair growth cycle.. Genes Dev.

[35] Goppner D, Leverkus M (2011). Basal cell carcinoma: from the molecular understanding of the pathogenesis to targeted therapy of progressive disease.. Journal of Skin Cancer.

