# Supplementary material for: Tumor Site Immune Markers Associated with Risk for Subsequent Basal Cell Carcinomas
Source: PLoS One. 2011 Sep 29;6(9):e25160. doi: 10.1371/journal.pone.0025160 (PMC3182995; doi:10.1371/journal.pone.0025160)
Supplement: Table S1 — Additional sun exposure characteristics of the analysis sample (n = 138). (DOC) [file pone.0025160.s001.doc]

**Table S1: Additional sun exposure characteristics of the analysis sample (n=138)**

|  | | | **N** | **%** |
| --- | --- | --- | --- | --- |
| **Sun exposure / Skin Type** | | |  |  |
|  | Number of sunburns, lifetime | |  |  |
|  |  | 0 | 3 | (2%) |
|  |  | 1-9 | 28 | (20%) |
|  |  | 10-19 | 30 | (22%) |
|  |  | 20-29 | 24 | (17%) |
|  |  | 30-39 | 11 | (8%) |
|  |  | 40-49 | 6 | (4%) |
|  |  | 50-59 | 21 | (15%) |
|  |  | 60+ | 14 | (10%) |
|  |  | Unknown | 1 | (1%) |
|  | Number of sunburns that blistered, lifetime | | |  |
|  |  | 0 | 28 | (20%) |
|  |  | 1-9 | 74 | (54%) |
|  |  | 10-19 | 18 | (13%) |
|  |  | 20+ | 18 | (13%) |
